# Magnetocardiography for identification of coronary ischemia in patients with chest pain and normal resting 12‐lead electrocardiogram

**DOI:** 10.1111/anec.12715

**Published:** 2019-10-06

**Authors:** Raja Ramesh, Sengottuvel Senthilnathan, Santhosh Satheesh, Pragyna Parimita Swain, Rajesh Patel, Ajith Ananthakrishna Pillai, Gireesan Katholil, Raja J. Selvaraj

**Affiliations:** ^1^ Department of Cardiology Jawaharlal Institute of Postgraduate Medical Education and Research Puducherry India; ^2^ Magnetoencephalography Laboratory, SQUIDs and Applications Section Materials Science Group Indira Gandhi Centre for Atomic Research Kalpakkam India

**Keywords:** basic, clinical, electrocardiography, non‐invasive techniques

## Abstract

**Background:**

Identification of coronary ischemia in patients presenting with chronic chest pain is difficult as resting ECG can be normal. Diagnosis of coronary ischemia requires evaluation during exercise or pharmacological stress. A noninvasive test to identify coronary ischemia at rest without the need for exercise is desirable. We studied the diagnostic accuracy of magnetocardiography (MCG) at rest to detect coronary ischemia in these patients.

**Methods:**

Patients with chronic chest pain and suspected coronary ischemia with a normal ECG were included. Patients underwent treadmill test (TMT) and were divided into TMT positive and TMT negative groups. MCG was recorded in a magnetically shielded room. Iso‐field contour maps generated at the T‐wave peak were compared between the groups. From the magnetic field map (MFM), the magnetic field angle at T‐wave peak was calculated and was also compared across the two groups.

**Results:**

There were a total of 29 patients, 12 with positive TMT and 17 with negative TMT. An abnormal magnetic field angle was more common in the TMT positive group (72% vs. 6%). Abnormal contour maps in the form of nondipole patterns or abnormal orientation were seen in 81.8% (9/11) patients in TMT positive group and 6.8% (1/17) patients in the TMT negative group (*p* < .001).

**Conclusion:**

Abnormal magnetic field angle and abnormal magnetic field maps in MCG recorded at rest are able to identify the presence of coronary ischemia in patients with chronic chest pain and a normal resting ECG.

## 1 INTRODUCTION

Chest pain is a common presentation of coronary ischemia. However, chest pain may also be a nonspecific symptom, unrelated to coronary ischemia. It is important to be able to differentiate these when a patient presents with chest pain. As resting electrocardiogram (ECG) is often normal in patients presenting with chest pain, diagnosis is usually made by identifying ECG changes of ischemia during exercise stress. This, however, requires the patient to be able to exercise sufficiently to raise the heart rate. Some patients are unable to exercise and pharmacological methods also have limitations. In such patients, other investigations like pharmacological stress with echocardiographic, MRI, or SPECT imaging is done, but these are cumbersome and resource intense. A noninvasive diagnostic test which is more sensitive than the ECG to identify coronary ischemia even at rest is hence desirable.

Magnetocardiography (MCG) is a technique which records the magnetic fields generated by the electrical activity of the heart using SQUID (superconducting quantum interference device) sensors (Fenici, Brisinda, & Meloni, 2005; Yamada & Yamaguchi, 2005). MCG has been extensively studied for the detection of fetal arrhythmias and noninvasive recording of his signal (Senthilnathan et al., 2017; Tavarozzi et al., 2002). It has also been found to be sensitive in identifying coronary ischemia as the tangential injury currents of myocardial ischemia are better picked by MCG than ECG (Gapelyuk et al., 2007).

This study was designed to assess MCG findings in patients with chest pain and a normal ECG and correlate these with the results of a treadmill test (TMT). We used treadmill test findings instead of coronary angiographic findings because it provides information on the functional significance of coronary artery stenosis and the presence of ischemia.

## 2 METHODS

### 2.1 Subjects

This was a prospective study performed among patients attending the Cardiology OPD in a tertiary care hospital. Patients with chronic chest pain and a normal resting 12 lead ECG were included. ECG was considered as normal if there were no pathologic Q waves and no changes in the ST segment or in T‐wave suggestive of ischemia. Patients with known coronary artery disease or prior myocardial infarction, atrial fibrillation or bundle branch block, crescendo or recent onset chest pain and those with high‐risk findings on TMT such as hypotension and ST elevation necessitating urgent angiography were excluded. Informed consent was obtained from all the patients, and the study protocol was approved by the Institute Ethics Committee.

### 2.2 TMT

All the patients underwent the TMT using the Bruce protocol with continuous monitoring of blood pressure, heart rate, and ST‐T changes in a 12 lead ECG. Patients with a positive TMT were considered as cases while those with a negative TMT formed the controls. TMT was labeled as positive if the ST segment showed 1 mm or greater, horizontal or downsloping depression at 60 milliseconds after the end of the QRS complex at peak exercise. TMT was labeled negative if the patient achieved at least 85% of target heart rate, and there were no ST changes. Patients with an inconclusive TMT for any reason were excluded.

### 2.3 MCG

**Figure 1:**
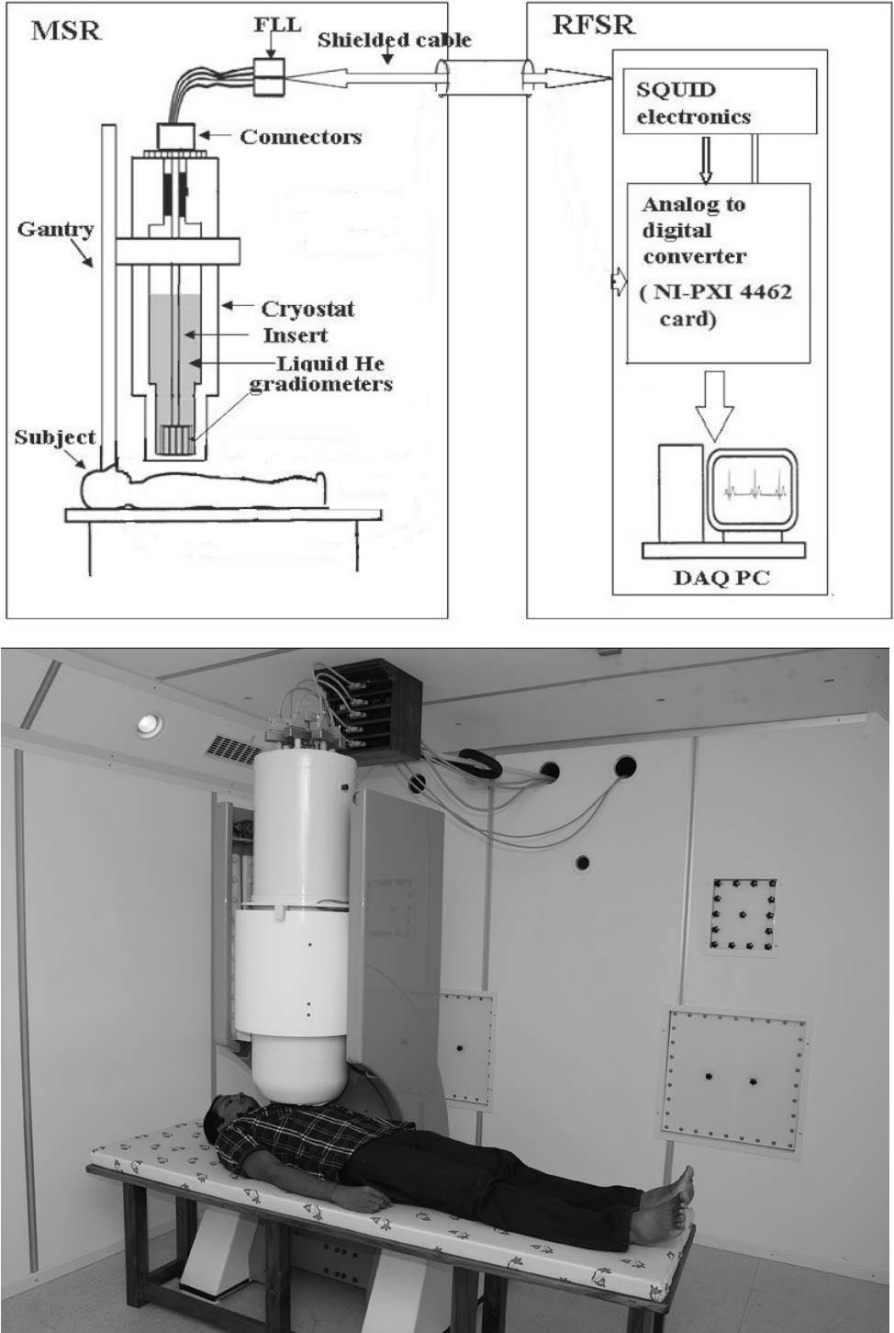
Magnetocardiogram recording. Top panel shows a schematic representation of the MCG recording setup. Bottom panel shows the array of SQUID sensors positioned over the chest of a supine patient for recording the MCG. DAQ PC, data acquisition system PC; FLL, flux‐locked loop; MSR, magnetically shielded room; NI PXI, National Instruments PCI eXtensions for Instrumentation; RFSR, radio frequency shielded room; SQUID, superconducting quantum interference device

The magnetocardiogram was recorded in a two‐layered magnetically shielded room using a 37‐channel MCG system consisting of an hexagonal array of direct current biased low transition temperature SQUID sensors operating at a temperature of 4.2 K provided by liquid helium (Parasakthi et al., 2012). The recording was done with the patient lying supine at rest. The signals were recorded from the front of chest keeping the MCG sensor grid as close as possible to chest but without direct contact (Figure 1). In order to get a comparable position of sensors in all patients, a chart marked with the position of the channels was attached to the front of the chest with reference to the sternal notch above and xiphoid below. The positioning of patient below SQUID was done to align this with a similar chart attached to the bottom of the SQUID cryostat containing liquid helium.

The MCG recording was performed with a bandwidth setting of 0–300 Hz at sampling rate of 1,000 Hz for a period of 5 min. The analog signals were digitized using an analog‐to‐digital converter with a resolution of 24 bits and stored in a server‐class personal computer for archival and offline data processing. Baseline wander imposed on the measured MCG signals due to body movements and respiration was corrected using wavelet transform technique. Wavelet “dB 10” with 10–12 levels of decomposition was performed to identify drifts and offset variations to subtracted from the as‐measured raw MCG data to remove baseline wander. The baseline‐corrected time series data were then subjected to independent component analysis (ICA) to demarcate cardiac signals and noise. Fast ICA algorithm was used to decompose MCG time series containing observations from 37 locations on the chest (Hyvarinen, 1999). Typically, twelve independent components were identified among which 5–7 were cardiac components while the rest were artifacts due to power line interference, vibration and other high‐frequency noise. The ICA cleaned MCG traces were superposed with the raw MCG data to ensure that no distortion was introduced during signal denoising. The ICA cleaned data were subjected to trigger locked averaging based on the time instants of R wave peaks (Sengottuvel, 2012). About 200 cardiac cycles were averaged to get one representative MCG beat from each measurement location. The averaged signal was smoothed using a moving window with 12 sample points to further refine the signal‐to‐noise ratio.

The averaged MCG signals of all 37 channels were further averaged across channels to extract a single MCG waveform which was used to automatically identify the ST segment. The QRS offset was algorithmically inferred by converting the MCG trace to a Gaussian curve by differentiation, squaring, and over smoothing (Senthilnathan et al., 2017, Senthilnathan 2012). QRS offset is then determined by the intersection to the x‐axis of a tangent which passes through the point of maximum slope of this curve. The T‐wave peak was identified, and the duration from the QRS offset to the T‐wave peak was divided into four equal segments. Magnetic field maps (MFM) were generated by plotting iso‐field contour maps at each of these four segments. A clear dipolar distribution was identified when a single maximum and minimum pole could be made out. In the absence of a clear dipole or when more than two poles were present, the map was labeled as showing a nondipole pattern. Maps were also considered to show an abnormal pattern when the poles were compressed, stretched, broken, or rotated. The magnetic field angle was measured as the angle of a line joining the negative and the positive poles in the MFM with the reference line.

### 2.4 Coronary angiogram

All the patients who had a positive TMT underwent coronary angiography and were classified as normal, single‐vessel disease, double‐vessel disease, or triple‐vessel disease depending on number of vessels showing significant (>70%) stenosis.

### 2.5 Statistical analysis

Demographic variables were compared between the groups using unpaired *t* test and chi‐square test for continuous and categorical variables, respectively. Discriminatory parameters from MCG recording were quantified and compared between the two groups with *t* test or chi‐square test. All the *t* tests were two sided, and a *p* value of .05 was considered significant.

## 3 RESULTS

### 3.1 Baseline characteristics

A total of 29 patients were recruited in the study, 12 with a positive TMT and 17 with negative TMT. Mean age of the patients was 53.2 years, and 79.3% were male. The baseline characteristics of the two groups are shown in Table 1.

**Table 1:** Baseline characteristics of patients

	TMT positive(*N* = 12)	TMT negative (*N* = 17)	*p* value
Male sex (%)	66%	88%	.19
Age (mean ± *SD*)	57.5 ± 7.2	50.18 ± 11.9	.25
Diabetes (%)	4 (33%)	4 (33%)	
Hypertension (%)	3 (25%)	6 (35%)	
LVH in ECHO	4 (33%)	3 (17.6%)	

### 3.2 MCG

**Figure 2:**
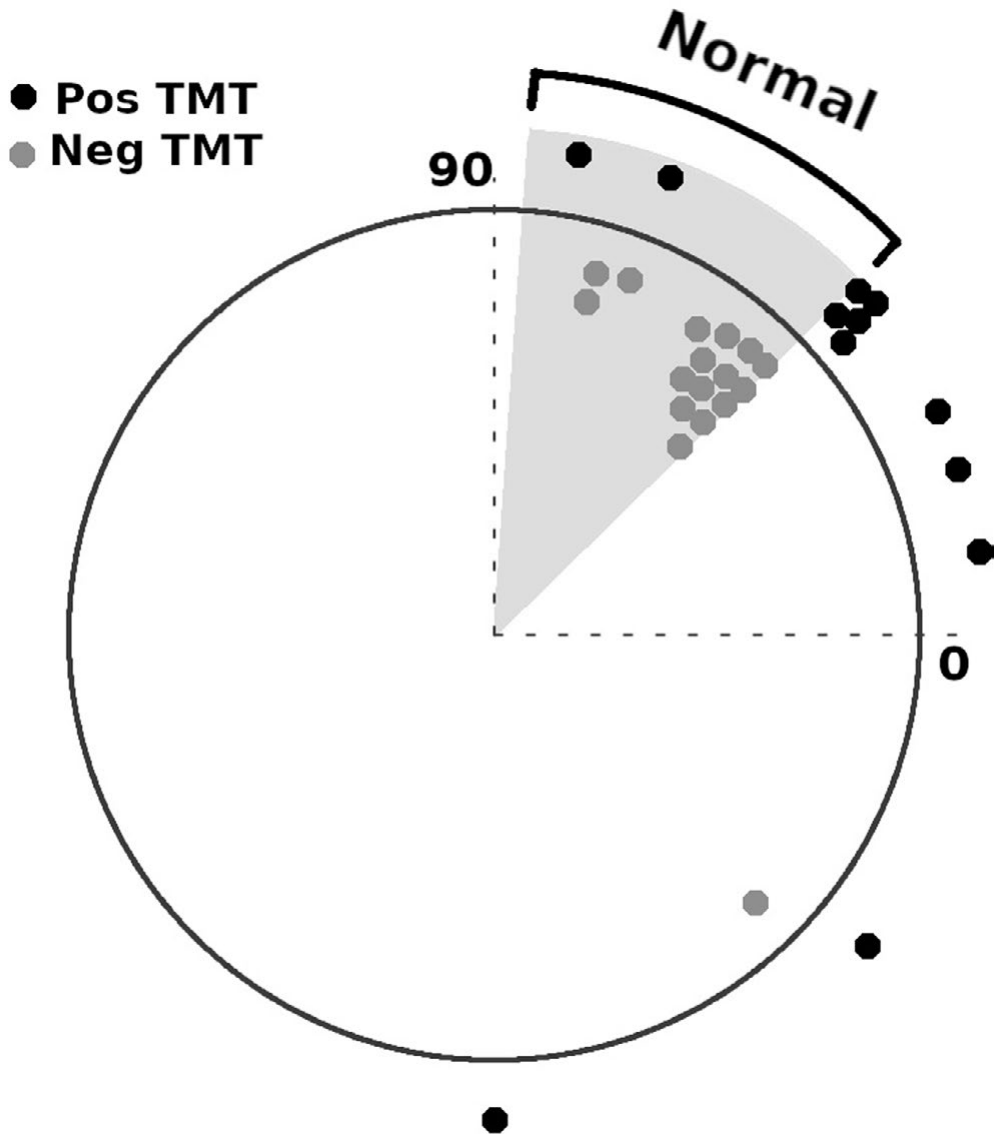
Magnetic field map angle at T‐wave peak for all patients. Magnetic field map angles are plotted for patients with positive TMT (red circles) and negative TMT (green circles)

Magnetocardiograms were recorded in all patients, but one recording in a patient from the TMT positive group could not be analyzed because of excessive noise. So, 11 MCGs from the TMT positive group and 17 from the TMT negative group were available for analysis. Magnetic field angle was classified as normal if it was between −86° to −45° (Lim et al., 2009). In the TMT negative group, 16 patients (94.1%) showed a normal angle as compared to 2 patients (18%) in the TMT positive group (*p* value .002) (Figure 2).

**Figure 3:**
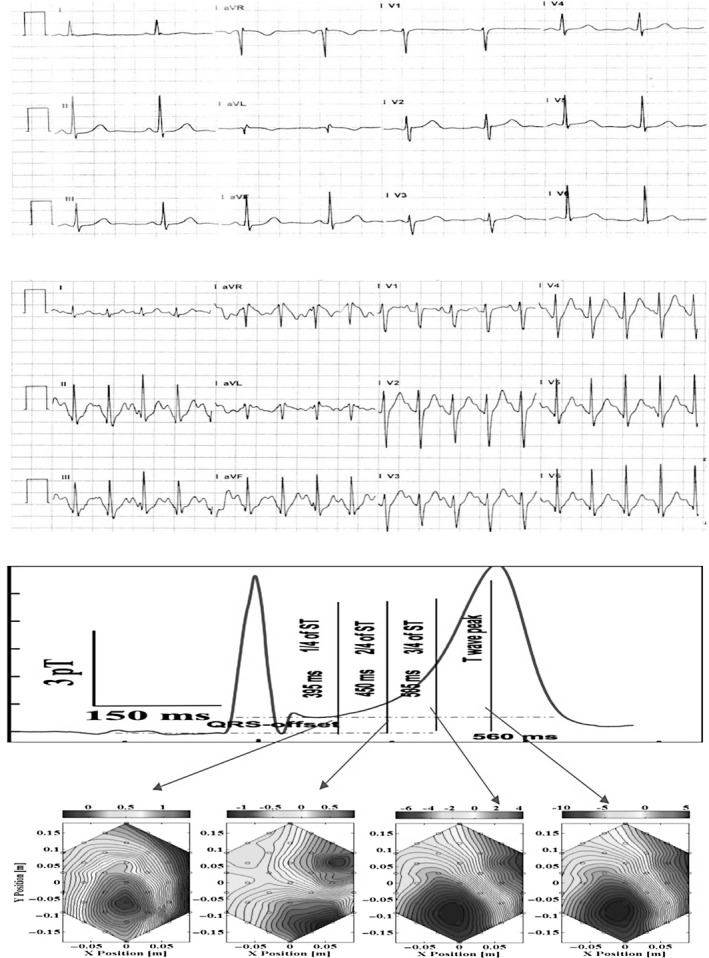
Magnetic field maps in a patient with negative TMT. Shown are the resting 12‐lead ECG (panel A), 12‐lead ECG during the stress test (panel B) and magnetic field maps at four time points on the ST segment (panel C). No ST changes are present at peak exercise. The magnetic field maps at all time points show a single dipole with normal orientation. The magnetic field angle map was –46°

**Figure 4:**
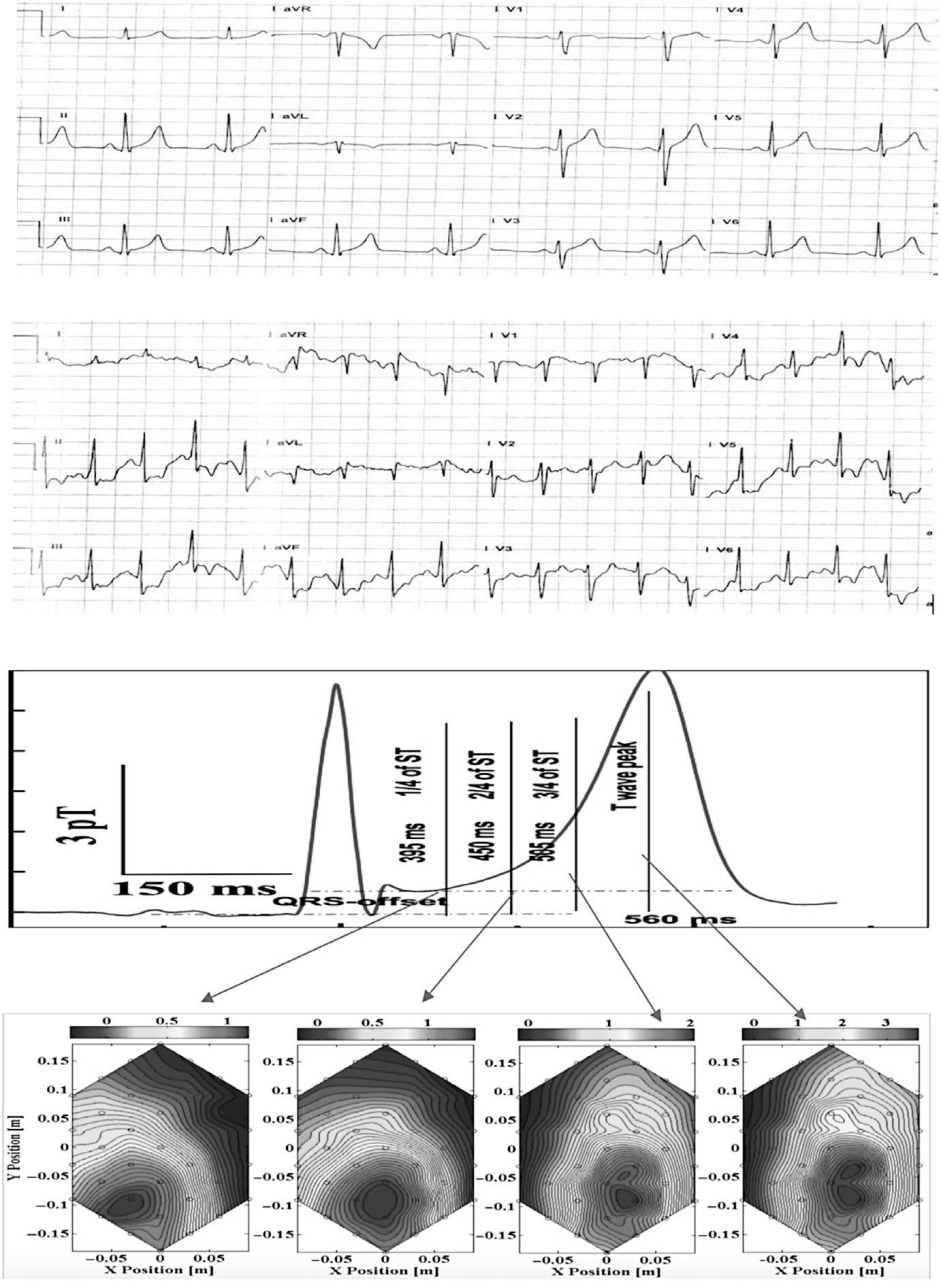
Magnetic field maps in a patient with positive TMT. Shown are the resting 12‐lead ECG (panel A), 12‐lead ECG during the stress test (panel B) and magnetic field maps at four time points on the ST segment (panel C). Significant ST depression is present at peak exercise indicating coronary ischemia. The magnetic field maps show a single pole which is stretched, abnormally oriented and split at the T‐wave peak. The magnetic field angle map was +40°

Abnormal contour maps at the T‐wave peak were seen in 9 of 11 (81.8%) patients in TMT positive group. Among these 9 patients, 7 showed abnormal orientation of the contour maps. The remaining two patients show more than one dipole either at the T‐wave peak or in the ST segment. Two patients with a positive TMT showed normal contour maps. In TMT negative group 16 of 17 (94.1%) patients showed normal contour maps at all time points on the ST segment, only one abnormal dipole pattern was observed. Figure 3 and Figure 4 show ECG under rest, ECG during peak treadmill stress and magnetic field maps in representative patients with normal TMT and abnormal TMT, respectively. Table 2 shows the sensitivity and specificity of abnormal map and abnormal angle to identify coronary ischemia.

**Table 2:** Sensitivity and specificity of MCG to identify coronary ischemia

	Sensitivity	Specificity
Abnormal angle	81.8%	94.1%
Abnormal map	81.8%	94.1%
Either map or angle abnormal	90.9%	94.1%

Among 11 TMT positive cases, 4 patients showed SVD, 3 showed DVD, and 1 showed TVD. Three patients with positive TMT and positive MCG had no significant stenosis on coronary angiogram.

## 4 DISCUSSION

This study of diagnostic accuracy showed that resting magnetocardiogram (MCG) has a high sensitivity and specificity in identifying coronary artery disease in patients with chronic chest pain even when the resting ECG was normal. Using two parameters, the presence of an abnormal magnetic field map and an abnormal magnetic field angle helps identify most patients with coronary ischemia.

We found that magnetic field angle was significantly different between the patients with coronary ischemia and normal patients. Most of the TMT negative patients had an angle within the normal range while it was outside this range in most of the TMT positive patients. Lim et al. found that the magnetic field angle was significantly different between normal subjects and patients presenting with acute coronary syndrome and no ST changes on ECG (Lim et al., 2009). Kwon et al. studied the usefulness of MCG in diagnosing coronary ischemia in suspected ACS patients without ST elevation in ECG. The magnetic field map angle at T‐wave maximum and maximum current angle were studied in these patients. These parameters of MCG showed sensitivity of 84% and specificity of 85% in diagnosing coronary ischemia in patients presenting with acute onset chest pain with normal ECG (Kwon et al., 2010)^.^

In our study, we also found that 81.8% subjects in TMT positive group showed abnormal contour maps and 94.1% patients in TMT negative group showed normal contour maps. Lim et al. found abnormal magnetic field maps in patients with 88% of patients with unstable angina and 93% of patients with non‐ST elevation myocardial infarction which is comparable to our study (Lim et al., 2009). Li et al., compared the MCG parameters between patients with coronary ischemia (angiographically identified coronary artery disease) and healthy controls. Abnormal MFM was seen in 74.6% of patients with coronary ischemia (Li et al., 2015). Shin et al. studied the diagnostic value of MCG to detect coronary ischemia based on nondipole magnetic field maps at T‐wave peak (Shin 2017). In their study, 84.8% of patients with coronary ischemia showed nondipole pattern in magnetic field maps while patients with no ischemia showed 88% normally oriented dipole maps (Shin et al., 2017).

We studied patients with suspected coronary ischemia, but with a normal resting ECG. It is in these patients that a new diagnostic modality like MCG carries significance. Previous studies have been done in patients with acute coronary syndromes where ECG changes are present at rest (Gapelyuk et al., 2007; Kwon et al., 2010; Li et al., 2015). In this setting, MCG corroborates the findings on the ECG, but there is no additional diagnostic value. In a study by Park et al (Park, Hill, Chung, Hugenholtz, & Jung, 2005), in patients with acute chest pain, many of whom did not have an abnormal ECG; MCG could identify the presence of coronary artery disease. Our study reinforces this finding and extends it to patients with chronic chest pain.

Coronary angiographic findings as used in previous studies (Gapelyuk et al., 2007; Hailer, Chaikovsky, Auth‐eisernitz, Schafer, & Van leeuwen, 2005; Kwon et al., 2010; Li et al., 2015; Lim et al., 2009; Shin et al., 2017; Wu et al., 2014) identify patients with coronary artery narrowing, but some of them may not be functionally significant. Instead, we classified patients into ischemic and nonischemic groups based on ECG changes during a treadmill test. ECG changes of ischemia during TMT identify patients with functionally important narrowing of the coronary arteries. Previously, Park et al (Park et al., 2015) also showed that MCG can also detect the functional significance of the coronary stenosis identified using fractional flow reserve.

Wu et al., and On et al., showed that resting MCG in chronic stable angina patients is more sensitive and specific than resting ECG in detecting coronary ischemia similar to our study although the parameters used were different (On et al., 2007; Wu et al., 2013).

Three patients with positive TMT and abnormal MCG had no significant stenosis in the epicardial coronary arteries. False positive TMT results are known to occur, typically in the presence of left ventricular hypertrophy or pre‐existing ST depression. However, in the absence of such abnormalities and in the presence of typical chest pain, a likely explanation for this is coronary ischemia due to microvascular dysfunction. The results obtained in the present study might possibly suggest that MCG in these patients could detect microvascular dysfunction as reported in a recent study (Shin, Park, & Lim, 2018).

### 4.1 Study limitations

The major limitation of our study is the small sample size. Although the results should be validated in a larger sample, we believe the findings are conclusive even in this sample. TMT has a sensitivity of 68% and specificity of 77% (Gibbons et al., 2002). This means there may be some false positive and false negatives. However, it has the advantage of identifying the presence of myocardial ischemia whereas coronary angiography does not give information on the functional significance of the lesion and its effect on myocardial perfusion. We used shielded MCG which provides better signal‐to‐noise ratio. However, with appropriate signal processing, MCG of good quality can be recorded even in an unshielded environment as reported by some researchers (Brisinda et al., 2003). Utility of unshielded MCG has to be studied as it can make it easier to perform the recording in a variety of situations.

## 5 CONCLUSIONS

The resting MCG analyzed using quantitative parameters (magnetic field angle) and qualitative parameters (nondipole phenomenon of magnetic field maps) has high diagnostic efficacy in detecting coronary artery disease among patients with chronic chest pain and a normal resting 12 lead ECG.

## CONFLICT OF INTEREST

None for any of the authors.
